# Patient Experiences of Receiving Stroke Discharge Information in Accordance With Preferences

**DOI:** 10.1111/jocn.17636

**Published:** 2025-01-09

**Authors:** Kristy Fakes, Amy Waller, Mariko Carey, Erin Forbes, Michael Pollack, Matthew Clapham, Robert Sanson‐Fisher

**Affiliations:** ^1^ Health Behaviour Research Collaborative, School of Medicine and Public Health, College of Health Medicine and Wellbeing University of Newcastle Callaghan New South Wales Australia; ^2^ Hunter Medical Research Institute New Lambton Heights New South Wales Australia; ^3^ Central Coast Research Institute, Central Coast Local Health District and University of Newcastle University of Newcastle Callaghan New South Wales Australia; ^4^ Hunter New England Local Health District New Lambton Heights New South Wales Australia; ^5^ Data Sciences Hunter Medical Research Institute New Lambton Heights New South Wales Australia

**Keywords:** cross‐sectional, discharge information, patient‐preferences, stroke, survey

## Abstract

**Aims:**

To examine survivors' experiences of discharge information including risk communication after hospitalisation for a stroke and the characteristics associated with receiving information in accordance with their preferences.

**Background:**

With advances in acute stroke care and an ageing population, the number of survivors of stroke is increasing. It is important that healthcare providers ensure patients have adequate information after a stroke‐related hospitalisation.

**Design:**

Cross‐sectional study.

**Methods:**

Adults recently discharged after a stroke from eight Australian hospitals were mailed a survey. Items examined risk and discharge care information, with participants asked to indicate both their preferences for and receipt of the information. Concordance with preferences was calculated, and characteristics associated with information preference concordance were assessed with binomial logistic regression. Study reported in accordance with STROBE Checklist.

**Results:**

Of 1161 eligible patients invited, 403 (35%) completed the survey. All items were endorsed by 80% or more of respondents as being wanted. However, for all items, fewer respondents reported the care as received. Only 28% of participants received information on all five items according to their preferences. Hospital site, Body Mass Index and age were statistically significantly associated with participants receiving information in accordance with their preferences.

**Conclusion:**

Most participants indicated a preference to receive recommended discharge information. Findings suggest that patients may benefit from increased information provision prior to hospital discharge after stroke.

**Relevance to Clinical Practice and Patient Care:**

Nurses have an important role in the provision of stroke care and information. The findings of this study may be used to improve the provision of post‐hospital discharge care and support for survivors of stroke, and assist in identifying patients at lower odds of experiencing information aligned with their preferences and who may benefit from support.

**Reporting Method:**

Strengthening the Reporting of Observational Studies in Epidemiology (STROBE) checklist for cross‐sectional studies.

**Patient or Public Contribution:**

No patient or public contribution.

**Summary**

- What does this paper contribute to the wider global clinical community?
  ○ Doctors and nurses need to consider the appropriate approach to align with patients' care goals. This study found hospital site, BMI and age statistically significantly associated with survivors of stroke receiving information in accordance with their preferences.
  ○ The odds for overweight and obese patients were 28% and 58%, respectively, less than for non‐overweight patients; and for every increase of 1 year of age, the odds of receiving discharge care information in accordance with their preference were 2% less.

## 1 Introduction

With advances in acute stroke care and an ageing population, the number of survivors of stroke is increasing. Nearly 90% of Australian survivors return home after their stroke (AIHW 2013), and the majority will require assistance with day‐to‐day living activities. As many survivors of stroke are living in the community, the responsibility for managing recovery will also likely be placed increasingly on the survivor and their support persons (Camak 2015; Crichton, Bray, and McKevitt 2016; Lin, Mei, and Wang 2021).

Nurses play an important role in stroke care. It is important that healthcare providers ensure patients are adequately prepared for what will happen during and after their stroke‐related hospital admission. The Australian and New Zealand Clinical Guidelines for Stroke Management recommend that information and education for stroke survivors should be offered at several different stages in the recovery journey (AIHW 2013; Crocker, Brown, and Lam 2021). However, there is increasing recognition that post‐discharge information and support are not consistently provided, with many expressing feelings of desertion once they are discharged from acute care. This sense of abandonment has been termed the ‘discharge chasm’ (Rudd 2006). Limited access to stroke services and community support services may exacerbate the challenges that survivors and their support persons face in managing their recovery.

## 2 Background

Communication of risk is essential to people recovering after a stroke. People who have experienced a transient ischaemic attack (TIA) or stroke are at much greater risk of a further stroke, with approximately 26% suffering another event within 5 years (Mohan, Wolfe, and Rudd 2011). However, it has been reported that between 10% and 46% of stroke survivors underestimate their risk of recurrent stroke (Boden‐Albala, Carman, and Moran 2011; Li, Tan, and Zhu 2022; Ren, Guo, and Zhang 2023; Saengsuwan and Suangpho 2019). Additionally, stroke is a highly preventable condition, and accurate communication of risk provides patients with the greatest chance to reduce their own modifiable risk factors (e.g., high blood pressure) (AIHW 2013). As such, it is important that people are aware of their level of risk, signs of another stroke and stroke prevention (AIHW 2013).

Relatively little is known about patients' preferences for risk and discharge care information post‐discharge after stroke. Most research has focused on mode of delivery. For example, a recent review found that a combination of written materials and discussions with healthcare providers was preferred by patients (Temehy, Rosewilliam, and Alvey 2022). A qualitative study conducted in Australia also found that survivors (*n* = 15) valued group education sessions (Finch, Minchell, and Cameron 2022). However, a survey of 34 acute stroke wards in Australia reported that only 15% of wards offered a group education programme, and while written materials and discussion with a healthcare provider were the most common mode of delivery, they were not necessarily delivered together (Hoffmann and Cochrane 2009). Further, 41.2% of the sites had reported that written materials were available ‘at patients request’.

Several intervention studies have been conducted examining the effectiveness of a range of strategies to improve patient education on stroke recovery (Benoit, Lopez, and Loiseau 2020; Crocker, Brown, and Lam 2021; Denny, Vahidy, and Vu 2017; Forster, Brown, and Smith 2012; Holzemer, Thanavaro, and Malmstrom 2011; Maasland, Koudstaal, and Habbema 2007; Olaiya, Cadilhac, and Kim 2017; Rodgers, Atkinson, and Bond 1999). However, there is limited research on preferences and receipt of information relating to post‐stroke risks and discharge information (Eames, Hoffmann, and Worrall 2011; Finch, Minchell, and Cameron 2022; Hoffmann and Cochrane 2009). Eames and colleagues explored patient preferences for information content prior to discharge (and 3 months post discharge), and found that all patients (*n* = 34) indicated a desire for clinical information prior to discharge (Eames, Hoffmann, and Worrall 2011). Other topics were less unanimously desired, however still preferred by most patients. No other studies have explored patients' preferences for information provision post‐discharge after stroke. Additionally, no research has examined whether information preferences have been met immediately post‐discharge. There is also a need to determine the factors associated with patients' experiences of discharge care. It would be useful to know which specific subgroups of patients may benefit from increased information prior to hospital discharge.

Consequently, this study aims to examine survivors' experiences of discharge care after hospitalisation for a stroke; and the sociodemographic, health and treatment characteristics associated with receiving information in accordance with their preferences for discharge care information.

## 3 Methods

### 3.1 Study Design

Cross‐sectional survey undertaken with survivors of stroke recruited from eight hospitals located in New South Wales (NSW). This article presents the discharge care‐related findings relating to a separate research question from a larger study that assessed patients' longer‐term unmet needs after stroke. This study is reported according to the Strengthening the Reporting of Observational Studies in Epidemiology (STROBE) Checklist of items that should be included in reports of cross‐sectional studies (Data S1).

### 3.2 Participants

Participants were recruited from eight hospitals located in NSW that provide inpatient acute and/or rehabilitation care to survivors of stroke. Both regional (*n* = 2) and major city (*n* = 6) hospitals were included. The study received institutional review board approval.

### 3.3 Eligibility

Eligible patients were those aged over 18 years, less than 3 months post first ischaemic or haemorrhagic stroke or transient ischaemic attack; and discharged home or to private rehabilitation, and able to provide informed consent, as indicated by return of the survey. Ineligible survivors were those assessed by the hospital stroke care coordinator/ward staff as having a severe neurological impairment not associated with stroke; severe language or cognitive impairment; or insufficient English to complete a survey.

### 3.4 Participant Recruitment and Data Collection

Clinic staff assessed patient eligibility at discharge. Eligible survivors of stroke were posted a study information and recruitment pack by clinic staff which contained an invitation to participate, information statement and pen‐and‐paper survey to return to the researchers via a reply‐paid envelope. Return of a completed survey was taken as implied consent. A reminder pack was sent to eligible patients approximately 2 weeks after the initial information pack.

### 3.5 Measures

#### 3.5.1 Quality of Discharge Care

Discharge care was assessed by five items developed from existing guidelines. These items were identified as important domains by consumers and consensus‐based national stroke guidelines (Stroke Foundation 2017). Respondents were asked five questions about their preferences for information (e.g., ‘At the time I was discharged from hospital, I would have liked information I could understand about: My potential risk of having another stroke in the next 12 months’). Patients were asked to indicate their preferences by selecting from response options: Strongly Agree, Agree, Disagree, Strongly Disagree, and Unsure. Participants were also asked about their receipt of information for the same five items (e.g., ‘At the time I was discharged from hospital, a healthcare provider gave me information in a way that I understood about: My potential risk of having another stroke in the next 12 months’). Response options: Strongly Agree, Agree, Disagree, Strongly Disagree, and Unsure. Additional items examined preferences and receipt of a discharge care plan.

#### 3.5.2 Demographic Characteristics

Age, level of education, employment status, and type of stroke were obtained via standard self‐report items. Treatment received was obtained by: ‘What medical treatments did you receive at the time of your stroke?’ with response options: ‘Thrombolysis (clot busting drug)’; ‘Carotid endarterectomy (surgery to remove plaque from the main artery in your neck)’; ‘Carotid angioplasty (surgery to place a stent in the main artery in your neck)’; ‘Endovascular clot retrieval (surgery to remove a clot from your brain)’; ‘Unsure’; and ‘Other’. Comorbid medical conditions were obtained by the item ‘Which of these health conditions apply to you?’, with response options: ‘History of Transient Ischaemic Attack (TIA)’; ‘Irregular Pulse (Atrial Fibrillation)’; ‘Diabetes’; ‘High blood pressure’; ‘High cholesterol’; ‘Smoker’; ‘Drink six or more standard alcoholic drinks per day’ and Body Mass Index (BMI) via two response options: BMI > 25 and BMI > 30, then re‐categorised into 3 categories: over 30 (Obese) for respondents who selected both BMI > 25 and BMI > 30 response options or BMI > 30; 26–30 (overweight) for respondents who selected only BMI > 25; and less than 25 for those who selected neither. This range of health and treatment‐related variables was included due to their potential utility in clinical settings.

#### 3.5.3 Statistical Analyses

Characteristics of participants were compared using means, standard deviations, medians and interquartile intervals for continuous variables and by percentages for categorical variables. For the items relating to discharge care information, participants who indicated a preference to receive any of the five items and also indicated they did receive that information were counted as having received the information in accordance with their preference. Pairwise complete cases were counted; that is, only non‐missing both preference and receiving items were counted, subjects missing items could still be counted. Subjects who did not want to receive information on all five items were excluded from the regression analysis, as it is not possible to receive any information in accordance with their preference. The associations of patient characteristics with receiving discharge care information in accordance with preferences were assessed with binomial logistic regression. Odds ratios with 95% confidence intervals and *p*‐values from likelihood ratio tests are provided.

#### 3.5.4 Sample Size

Assuming conservatively a true underlying proportion of participants reporting discharge care information of 0.5, a sample of *n* = 400 would be able to estimate this with a 5% Margin of Error using an exact Clopper‐Pearson Interval. Statistical analyses were programmed using R version 4.0.3 (2020‐10‐10) (R Foundation for Statistical Computing, Vienna, Austria. URL http://www.R‐project.org/).

### 3.6 Ethics

Ethics approvals were obtained from: Hunter New England Local Health District Human Research Ethics Committee (16/05/18/4.04; 2019/ETH03871); Mid North Coast Local Health District (HREC/16/HNE/190); South‐Western Sydney Local Health District (HE18/086); and Western Sydney Local Health District (5561) and University of Newcastle Human Research Ethics Committee (H‐2016‐0233).

## 4 Results

Recruitment and data collection took place from April 2018 to December 2019. A total of 1308 patients were assessed for study eligibility, of which 1140 were identified as being eligible. Reasons for ineligibility included: over 3 months post‐stroke (*n* = 3) and not discharged home (*n* = 165). Of the 1140 eligible patients who were posted a recruitment pack, 403 (35%) consented to participate and completed the survey. As shown in Table 1, most participants were hospitalised for an ischaemic stroke (41%).

**TABLE 1:** Demographic and health characteristics of participants (*N* = 403).

Patient characteristics	*n* (%)*
Sex	
Male	236 (59.6)
Female	160 (40.4)
Age (years)	
Mean (SD)	70.1 (12.3)
Age 18–44	11 (2.9)
Age 45–64	94 (24.4)
Age ≥ 65	280 (72.7)
Education	
Secondary school or below	200 (51.5)
Trade or vocational training (e.g., TAFE or college)	131 (33.8)
Tertiary	57 (14.7)
Working status	
Working (full, part time or casual)	95 (24.2)
Not working (home duties, unemployed, retired, disability pension)	297 (75.8)
Private health insurance	
Yes	189 (48.8)
Health and treatment characteristics	
Type of stroke	
Ischaemic stroke	157 (41.3)
Haemorrhagic stroke	20 (5.3)
Transient Ischaemic Attack (TIA)	109 (28.7)
Unsure	94 (24.7)
Medical treatments received for stroke	
Thrombolysis	112 (28.6)
Carotid endarterectomy	17 (4.3)
Carotid angioplasty	4 (1.0)
Endovascular clot retrieval	35 (9.0)
Unsure	146 (37.3)
Other (e.g., medication, other tests)	64 (16.4)
Other health conditions and lifestyle risk factors	
History of Transient Ischaemic Attack (TIA)	57 (14.8)
Irregular pulse (Atrial Fibrillation)	89 (23.1)
Diabetes	104 (27.0)
High blood pressure	208 (54.0)
High cholesterol	123 (31.9)
Smoker	51 (13.2)
Overweight (Body Mass Index over 25)	71 (18.4)
Obese (Body Mass Index over 30)	19 (4.9)
(Body Mass Index under 26)	300 (77.9)
(Body Mass Index 26–30)	66 (17.1)
(Body Mass Index over 30)	19 (4.9)
Drink six or more standard alcoholic drinks per day	22 (5.7)

* Observations within each variable may not add to total sample size due to missing values.

Table 2 presents the number and proportion of survivors who reported wanting information about recommended discharge care items. All five items were endorsed by 80% or more of respondents as being wanted, with endorsement ranging from 80% to 83%. Thirty‐two participants (7.9%) didn't want any of the five items of information, and 24 (6%) did not provide a response for all five of the preference items. Most participants (78%, *n* = 213) also indicated they would have liked a written discharge plan.

**TABLE 2:** Number and percentage of participants who reported wanting information about discharge care.

At the time I was discharged from hospital, I would have liked information I could understand about	Strongly agree *n* (%)	Agree *n* (%)	Disagree *n* (%)	Strongly disagree *n* (%)	Unsure *n* (%)
My potential risk of having another stroke in the next 12 months	159 (42.5)	141 (37.7)	24 (6.4)	8 (2.1)	42 (11.2)
How to prevent another stroke	155 (41.9)	141 (38.1)	22 (5.9)	6 (1.6)	46 (12.4)
Coping with common symptoms and side‐effects of stroke	135 (37.3)	165 (45.6)	30 (8.3)	7 (1.9)	25 (6.9)
Who I should contact if I had any stroke‐related questions or concerns	138 (38.1)	152 (42.0)	32 (8.8)	12 (3.3)	28 (7.7)
When I should contact someone about stroke‐related signs and symptoms	145 (40.1)	148 (40.9)	32 (8.8)	10 (2.8)	27 (7.5)

### 4.1 Perceived Quality of Discharge Care

Table 3 presents the number and proportion of survivors who reported receiving information about recommended discharge care items. Gaps in patient‐centred care were identified for all five items; with all five items having less than 80% of respondents reporting the information as received. Approximately one third of participants reported they did not receive the recommended discharge information. This ranged from 120 participants (32%) reporting they did not receive information on who they should contact if they had any stroke‐related questions or concerns to 38% of participants (*n* = 146) reporting that they did not receive information in relation to their potential risk of having another stroke in the next 12 months. In addition, one in five participants reported not receiving a written discharge plan (yes = 72%, unsure = 9%).

**TABLE 3:** Number, proportion survivors receiving information about discharge care (*N* = 403).

	Patient preferences for information: endorsed ‘strongly agree’ or ‘agree’		Information received: ‘strongly agree’ or ‘agree’ responses	
	*n*	(*n*, %)	*n*	(*n*, %)
My potential risk of having another stroke in the next 12 months	374	300 (80%)	382	185 (48%)
How to prevent another stroke	370	296 (80%)	381	192 (50%)
Coping with common symptoms and side‐effects of stroke	362	300 (83%)	377	192 (51%)
Whom I should contact if I had any stroke‐related questions or concerns	362	290 (80%)	377	217 (58%)
When I should contact someone about stroke‐related signs and symptoms	362	293 (81%)	378	210 (56%)

*Note:* Patients' preferences and whether item was received in relation to their discharge care.

In relation to whether participants received the information in accordance with their preferences, the mean number of items participants reported receiving according to their preferences was 2.3 (SD: 2.1), and the median was 2.0 (IQR: 5.0). The total number of participants who received information on all five items according to their preferences was *n* = 93 (27.7%). Twenty‐nine participants (8.6%) received information according to their preferences for four of the five items; 27 (8.0%) received it for three items; 33 (9.8%) for two items; and 44 (13.1%) for one item only. There were 110 participants (32.7%) who did not receive information according to their preferences for any of the five items.

Table 4 presents the regression results for receiving discharge care information in accordance with preferences. These are the factors associated with receiving information in accordance with their preferences for at least one of the five discharge care items. Hospital site, BMI, age and type of stroke were significantly associated with receipt of care aligned with preferences. The odds varied between hospitals for receipt of discharge care information according to preferences. The odds for overweight patients receiving discharge care information in accordance with their preference were 28% less than for non‐overweight patients, OR: 0.72 (95% CI: 0.53–0.98). For obese, 58% less OR: 0.42 (95% CI: 0.25–0.72). For every increase of 1 year of age, the odds of receiving discharge care information in accordance with their preference were 2% less, OR: 0.98 (95% CI: 0.97–1).

**TABLE 4:** Binomial regression results for outcome receiving discharge care information in accordance with their preferences.

Variable	Unadjusted OR (95% CI)	*p*	Adjusted OR (95% CI)	*p*
Sociodemographic characteristics				
Gender				
Male	Reference	0.255	Reference	0.997
Female	1.13 (0.91–1.40)		1.00 (0.78–1.28)	
Age				
Continuous variable	0.99 (0.98–1.00)	0.123	0.98 (0.97–1.00)	0.007
Employment status				
Not working (home duties, retired, unemployed, disability pension)	Reference	0.562	Reference	0.498
Working (full, part time or causal)	1.07 (0.85–1.36)		1.12 (0.80–1.57)	
Private health insurance				
No	1.10 (0.90–1.36)	0.356	0.88 (0.68–1.15)	0.344
Health and treatment characteristics				
Treatment centre				
Hospital 1 (major city)	Reference	< 0.001	Reference	< 0.001
Hospital 2 (major city)	1.99 (0.96–4.30)		1.79 (0.83–4.02)	
Hospital 3 (major city)	2.51 (1.28–5.15)		2.84 (1.33–6.26)	
Hospital 4 (inner regional)	3.09 (2.21–4.36)		3.73 (2.48–5.68)	
Hospital 5 (inner regional)	1.46 (0.96–2.22)		1.50 (0.96–2.35)	
Hospital 6 (major city)	1.50 (1.10–2.04)		1.36 (0.97–1.91)	
Hospital 7 (major city)	0.67 (0.45–0.99)		0.50 (0.32–0.77)	
Hospital 8 (major city)	2.08 (1.46–2.98)		2.04 (1.35–3.10)	
Type of stroke				
Ischaemic stroke	Reference	0.063	Reference	0.012
Haemorrhagic stroke	0.69 (0.41–1.13)		0.69 (0.39–1.21)	
Transient Ischaemic Attack (TIA)	1.12 (0.86–1.45)		0.72 (0.53–0.99)	
Unsure	1.30 (0.99–1.70)		1.27 (0.92–1.76)	
Medical treatments received for stroke				
Thrombolysis (=yes)	0.86 (0.68–1.08)	0.184	0.80 (0.60–1.05)	0.105
Other health conditions and lifestyle risk factors				
History of Transient Ischaemic Attack (TIA)	1.70 (1.26–2.31)	< 0.001	1.41 (1.00–1.99)	0.052
Irregular pulse (Atrial Fibrillation)	1.14 (0.89–1.46)	0.288	1.02 (0.75–1.38)	0.906
Diabetes	0.92 (0.73–1.15)	0.454	1.00 (0.75–1.33)	0.983
High blood pressure	1.05 (0.85–1.30)	0.641	1.27 (0.98–1.66)	0.069
High cholesterol	0.92 (0.73–1.14)	0.438	1.05 (0.80–1.37)	0.717
Smoker	0.88 (0.64–1.22)	0.439	1.02 (0.68 1.51)	0.939
BMI				
BMI under 26	Reference	0.017	Reference	0.002
Overweight (BMI 26–30)	0.74 (0.57–0.97)		0.72 (0.53–0.98)	
Obese (BMI over 30)	0.60 (0.37–0.97)		0.42 (0.25–0.72)	

*Note:* Regressions use all available cases, multivariable regression *N* = 291.

## 5 Discussion

This study is the first to assess the extent to which the delivery of discharge information meets the preferences of patients and the factors associated with this. All five items were endorsed by 80% or more of respondents as being wanted, with endorsement ranging from 80% to 83%. A cut point of 80% is a standard approach for measuring consensus (Diamond, Grant, and Feldman 2014). However, whilst endorsement was over 80%, for all five items, fewer respondents (< 80%) reported the care as received. Only 27.7% (*n* = 93) of participants received information on all five items according to their preferences, and the mean number of items that participants reported receiving according to their preferences was 2.3 (SD: 2.1). Our findings suggest that there is a gap in the provision of patient‐centred discharge care information which should be addressed. These findings suggest patients may benefit from increased information provision prior to hospital discharge after stroke. This is essential, as a key feature of patient‐centred care is the integration of patient preferences in all aspects of healthcare, including the communication of information (Pelzang 2010). Further, research indicates that poor‐quality discharge care is associated with greater levels of long‐term unmet needs among survivors (Andrew, Busingye, and Lannin 2018).

Healthcare providers need to communicate accurate health information to patients. Nurses have an important role in the provision of information relating to the patient's care, risk, including secondary prevention management, and discharge care information. It is acknowledged that this can be challenging as outcomes after a stroke can be variable with recovery profiles varying greatly between survivors (Grefkes and Fink 2020). However, it is concerning that 35%–38% of participants did not receive information about their potential risk of having another stroke in the next 12 months, how to prevent another stroke, common symptoms and side‐effects of stroke. These findings are consistent with a recent study of stroke survivors (*n* = 200) who reported the overall quality of discharge care planning as subpar (Andrew, Busingye, and Lannin 2018).

In our study, most participants (78%) also indicated they would have liked a written discharge plan, and 72% reported receiving a written discharge plan. This figure aligns closely with recently reported Australian figures, which indicate 70% of people in Acute Services had a care plan developed (Stroke Foundation. 2023). The Australian Clinical Practice Guidelines for Stroke Management detail a strong recommendation that ‘comprehensive discharge care plans that address the specific needs of the stroke survivor should be developed in conjunction with the stroke survivor and carer prior to discharge’. (Stroke Foundation 2023). These findings highlight that there is room for improvement in the provision of care plans. The large number of sites included in this study enabled the association between hospital site, in addition to sociodemographic and health and treatment characteristics, to be examined for the first time in relation to patients receiving discharge care information in accordance with preferences. After controlling for other variables, hospital site, BMI and age were significantly associated with patients receiving discharge care information in accordance with preferences.

The results varied between hospitals. The odds were higher in Hospital 3 (major city), Hospital 4 (inner regional) and Hospital 8 (major city) compared to Hospital 1 (major city), with odds ratios for those hospitals ranging from 2.04 to 3.73. The odds were 50% less in Hospital 7 (major city), OR: 0.50 (95% CI: 0.32–0.77). Other research in relation to hospital location has been published by others. The 2021 National Stroke Audit (Stroke Foundation 2021) reported that hospitals in more remote locations were much less likely to provide important discharge information, and provision of information regarding secondary prevention was also poorer in regional services, compared to major cities. Inconsistency in the provision of information is in line with other previous research that suggests significant variation between acute stroke wards. According to a study of 34 acute stroke wards in Australia, only 55% of sites provided information post‐discharge (Hoffmann and Cochrane 2009).

Body Mass Index was also strongly associated with receipt of discharge care information. The odds for overweight patients receiving discharge care information in accordance with their preference were 28% less than for non‐overweight patients, OR: 0.72 95% CI: 0.53–0.98. For obese patients, the odds for receiving discharge care information in accordance with their preferences were 58% less OR: 0.42 95% CI: 0.25–0.72. This is an important finding, given obesity is a potentially modifiable risk factor for stroke. The finding that a higher BMI was associated with lower odds or receiving information according to preferences has not previously been reported in relation to care after a stroke. However, the impact of weight bias and stigma on quality of care has been systematically reviewed, with considerable evidence for the impact of negative attitudes and stereotypes found to influence perceptions and decision‐making for people with obesity (Phelan et al. 2015). This may be one possible explanation for study findings. However, it would not be expected that healthcare providers would intentionally discriminate against patients with a higher BMI. It is suggested these aspects, including any healthcare provider assumptions about patient needs, be examined in future research.

For every increase of 1 year of age, the odds of receiving discharge care information in accordance with their preference were 2% less, OR: 0.98 95% CI: 0.97–1. These findings support other research which has suggested older adults are significantly less involved in their medical decisions than younger adults (Zikmund‐Fisher, Couper, and Fagerlin 2012). Additionally, challenges with delivering person‐centred care to older patients, including cognitive impairment and hearing loss have been highlighted in the literature (Nilsen, Hollister, and Söderhamn 2022).

### 5.1 Strengths and Limitations

This was a large study conducted with patients recruited from eight hospitals. However, there are several study limitations. Data on medical conditions (e.g., blood pressure, cholesterol) were obtained via participant self‐report only. The accuracy of this information was not determined for this study. These results may not be generalisable to survivors with more severe cognitive impairment or aphasia, or survivors discharged to residential care, as these sub‐groups were excluded from the study. Further, we excluded those with insufficient English to complete a survey. It is possible that other characteristics than those examined in this study may influence both the care patients receive, and their perceptions of the care they receive, such as education and self‐efficacy, health literacy, and cultural and linguistic background. While we did not examine these factors in the current study, they would be a worthy focus of ongoing research. Future research should also examine the format of information provided. Other research has highlighted potential ways that information provision can be improved. For example, a UK study that examined the effectiveness of active and passive information provision for stroke found that active information provision may improve stroke‐related knowledge (Crocker, Brown, and Lam 2021). A US study has also reported that information provided by video improved patients' stroke knowledge, self‐efficacy in recognising stroke symptoms, and satisfaction with their stroke education (Denny, Vahidy, and Vu 2017).

### 5.2 Relevance to Clinical Practice

Study findings may be used to make changes to improve the post‐hospital stroke discharge care and support provided to survivors. As patients' preferences regarding information preferences may differ, doctors and nursing staff need to consider the appropriate approach to align with patients' care goals. Some characteristics could be easily identified (e.g., age, BMI) prior to the patient's discharge from hospital.

## 6 Conclusion

Most participants indicated a preference to receive recommended discharge information. Hospital site, BMI and age were significantly associated with survivors of stroke receiving discharge care information in accordance with preferences. Together, these findings assist in identifying patients at lower odds of experiencing information aligned with their preferences and who may benefit from support.

## Author Contributions

Amy Waller, Mariko Carey, Michael Pollack and Robert Sanson – Fisher contributed to funding acquisition and study conceptualisation. Kristy Fakes, Amy Waller, Mariko Carey and Rob Sanson – Fisher contributed to investigation, methodology, project management, supervision. Kristy Fakes, Amy Waller, Mariko Carey, Erin Forbes, Matthew Clapham and Michael Pollack contributed to writing: original draft manuscript. Matthew Clapham contributed to data curation and formal analysis. All authors reviewed and edited the manuscript and approved the final version of the manuscript.

## Conflicts of Interest

The authors declare no conflicts of interest.

## Supporting information


Data S1.

## Data Availability

The data are not publicly available due to ethical restrictions. Additional use of or access to the data requires that the research team submit a request for variation of ethics approval.

## References

[jocn17636-bib-0001] AIHW . 2013. Stroke and Its Management. An Update. (Cardiovascular disease series no. 37. Cat. no. CVD 61. (Canberra: Australian Institute of Health and Welfare, 2013).

[jocn17636-bib-0002] Andrew, N. E. , D. Busingye , and N. A. Lannin . 2018. “The Quality of Discharge Care Planning in Acute Stroke Care: Influencing Factors and Association With Post‐Discharge Outcomes.” Journal of Stroke and Cerebrovascular Diseases 27: 583–590. 10.1016/j.jstrokecerebrovasdis.2017.09.043. 29097058

[jocn17636-bib-0003] Benoit, C. , D. Lopez , and M. Loiseau . 2020. “Impact of a Pre‐Discharge Education Session on Stroke Knowledge: A Randomized Trial.” Journal of Stroke and Cerebrovascular Diseases 29: 105272. 10.1016/j.jstrokecerebrovasdis.2020.105272. 32992206

[jocn17636-bib-0004] Boden‐Albala, B. , H. Carman , and M. Moran . 2011. “Perception of Recurrent Stroke Risk Among Black, White and Hispanic Ischemic Stroke and Transient Ischemic Attack Survivors: The SWIFT Study.” Neuroepidemiology 37: 83–87. 10.1159/000329522. 21894045 PMC3186718

[jocn17636-bib-0005] Camak, D. J. 2015. “Addressing the Burden of Stroke Caregivers: A Literature Review.” Journal of Clinical Nursing 24: 2376–2382. 10.1111/jocn.12884. 26095074

[jocn17636-bib-0006] Crichton, S. L. , B. D. Bray , and C. McKevitt . 2016. “Patient Outcomes up to 15 Years After Stroke: Survival, Disability, Quality of Life, Cognition and Mental Health.” Journal of Neurology, Neurosurgery, and Psychiatry 87: 1091–1098. 10.1136/jnnp-2016-313361. 27451353

[jocn17636-bib-0007] Crocker, T. F. , L. Brown , and N. Lam . 2021. “Information Provision for Stroke Survivors and Their Carers.” Cochrane Database of Systematic Reviews 11: 001919. 10.1002/14651858.CD001919.pub4. PMC8610078 34813082

[jocn17636-bib-0008] Denny, M. C. , F. Vahidy , and K. Y. Vu . 2017. “Video‐Based Educational Intervention Associated With Improved Stroke Literacy, Self‐Efficacy, and Patient Satisfaction.” PLoS One 12: 0171952. 10.1371/journal.pone.0171952. PMC5364024 28333925

[jocn17636-bib-0009] Diamond, I. R. , R. C. Grant , and B. M. Feldman . 2014. “Defining Consensus: A Systematic Review Recommends Methodologic Criteria for Reporting of Delphi Studies.” Journal of Clinical Epidemiology 67: 401–409. 10.1016/j.jclinepi.2013.12.002. 24581294

[jocn17636-bib-0010] Eames, S. , T. Hoffmann , and L. Worrall . 2011. “Delivery Styles and Formats for Different Stroke Information Topics: Patient and Carer Preferences.” Patient Education and Counseling 84: 18–23. 10.1016/j.pec.2010.07.007. 20708899

[jocn17636-bib-0011] Finch, E. , E. Minchell , and A. Cameron . 2022. “What Do Stroke Survivors Want in Stroke Education and Information Provision in Australia?” Health & Social Care in the Community 30: 20220629. 10.1111/hsc.13896. PMC10084245 35768909

[jocn17636-bib-0012] Forster, A. , L. Brown , and J. Smith . 2012. “Information Provision for Stroke Patients and Their Caregivers.” Cochrane Database of Systematic Reviews 11: 001919. 10.1002/14651858.CD001919.pub3. PMC6544775 23152210

[jocn17636-bib-0013] Grefkes, C. , and G. Fink . 2020. “Recovery From Stroke: Current Concepts and Future Perspectives.” Neurological Research and Practice 2: 17. 10.1186/s42466-020-00060-6. 33324923 PMC7650109

[jocn17636-bib-0014] Hoffmann, T. , and T. Cochrane . 2009. “What Education Do Stroke Patients Receive in Australian Hospitals?” Patient Education and Counseling 77: 187–191. 10.1016/j.pec.2009.03.009. 19356883

[jocn17636-bib-0015] Holzemer, E. M. , J. Thanavaro , and T. K. Malmstrom . 2011. “Modifying Risk Factors After TIA and Stroke: The Impact of Intensive Education.” Journal for Nurse Practitioners 7: 372–377. 10.1016/j.nurpra.2011.01.017.

[jocn17636-bib-0016] Li, J. , J. Tan , and F. Zhu . 2022. “Comparisons of Stroke Knowledge and Health Behaviors in Patients With Hypertensive Stroke at Different Recurrence Risk Strata: The Comprehensive Reminder System Based on the Health Belief Model Study.” Journal of Cardiovascular Nursing 37: 184–191. 10.1097/jcn.0000000000000765. 33605641

[jocn17636-bib-0017] Lin, B. , Y. Mei , and W. Wang . 2021. “Unmet Care Needs of Community‐Dwelling Stroke Survivors: A Systematic Review of Quantitative Studies.” BMJ Open 11: 45560. 10.1136/bmjopen-2020-045560. PMC8061855 33879490

[jocn17636-bib-0018] Maasland, E. , P. J. Koudstaal , and J. D. Habbema . 2007. “Effects of an Individualized Multimedia Computer Program for Health Education in Patients With a Recent Minor Stroke or Transient Ischemic Attack—A Randomized Controlled Trial.” Acta Neurologica Scandinavica 115: 41–48. 10.1111/j.1600-0404.2006.00722.x. 17156264

[jocn17636-bib-0019] Mohan, K. M. , C. D. Wolfe , and A. G. Rudd . 2011. “Risk and Cumulative Risk of Stroke Recurrence: A Systematic Review and Meta‐Analysis.” Stroke 42: 1489–1494. 10.1161/strokeaha.110.602615. 21454819

[jocn17636-bib-0020] Nilsen, E. R. , B. Hollister , and U. Söderhamn . 2022. “What Matters to Older Adults? Exploring Person‐Centred Care During and After Transitions Between Hospital and Home.” Journal of Clinical Nursing 31: 569–581. 10.1111/jocn.15914. 34117673

[jocn17636-bib-0021] Olaiya, M. T. , D. A. Cadilhac , and J. Kim . 2017. “Effectiveness of an Intervention to Improve Risk Factor Knowledge in Patients With Stroke.” Stroke 48: 1101–1103. 10.1161/STROKEAHA.116.016229. 28250198

[jocn17636-bib-0022] Pelzang, R. 2010. “Time to Learn: Understanding Patient‐Centred Care.” British Journal of Nursing 19: 912–917. 10.12968/bjon.2010.19.14.49050. 20647984

[jocn17636-bib-0023] Phelan, S. M. , D. J. Burgess , M. W. Yeazel , W. L. Hellerstedt , J. M. Griffin , and M. van Ryn . 2015. “Impact of Weight Bias and Stigma on Quality of Care and Outcomes for Patients With Obesity.” Obesity Reviews 16, no. 4: 319–326. 10.1111/obr.12266. 25752756 PMC4381543

[jocn17636-bib-0024] Ren, H. , Y. F. Guo , and Z. X. Zhang . 2023. “Perception of Recurrent Risk Versus Objective Measured Risk of Ischemic Stroke in First‐Ever Stroke Patients From a Rural Area in China: A Cross‐Sectional Study.” Patient Education and Counseling 107: 20221205. 10.1016/j.pec.2022.107586. 36495680

[jocn17636-bib-0025] Rodgers, H. , C. Atkinson , and S. Bond . 1999. “Randomized Controlled Trial of a Comprehensive Stroke Education Program for Patients and Caregivers.” Stroke 30: 2585–2591. 10.1161/01.str.30.12.2585. 10582982

[jocn17636-bib-0026] Rudd, A. G. 2006. “Stroke: Improving Transfer of Care Pathways‐While the Management of Stroke Care Has Improved, There Are Still Lapses in Communication Between Primary and Secondary Settings.” Geriatric Medicine‐Coventry 36, no. 11: 67–70.

[jocn17636-bib-0027] Saengsuwan, J. , and P. Suangpho . 2019. “Self‐Perceived and Actual Risk of Further Stroke in Patients With Recurrent Stroke or Recurrent Transient Ischemic Attack in Thailand.” Journal of Stroke and Cerebrovascular Diseases 28: 20181129. 10.1016/j.jstrokecerebrovasdis.2018.11.001. 30503679

[jocn17636-bib-0028] Stroke Foundation . 2017. Clinical Guidelines for Stroke Management.(Melbourne: Stroke Foundation, 2017).

[jocn17636-bib-0029] Stroke Foundation . 2021. National Stroke Audit—Acute Services Report. (Melbourne: Stroke Foundation, 2021).

[jocn17636-bib-0030] Stroke Foundation . 2023. “Clinical Guidelines for Stroke Management.” https://informme.org.au/guidelines/living‐clinical‐guidelines‐for‐stroke‐management.

[jocn17636-bib-0031] Temehy, B. , S. Rosewilliam , and G. Alvey . 2022. “Exploring Stroke Patients' Needs After Discharge From Rehabilitation Centres: Meta‐Ethnography.” Behavioral Science 12: 404. 10.3390/bs12100404 PMC9598696 36285973

[jocn17636-bib-0032] Zikmund‐Fisher, B. J. , M. P. Couper , and A. Fagerlin . 2012. “Disparities in Patient Reports of Communications to Inform Decision Making in the DECISIONS Survey.” Patient Education and Counseling 87: 198–205. 10.1016/j.pec.2011.08.002. 21890299

